# Extraction of Al from Coarse Al–Si Alloy by The Selective Liquation Method

**DOI:** 10.3390/ma14133680

**Published:** 2021-07-01

**Authors:** Bo Li, Yaowu Wang, Bingliang Gao

**Affiliations:** School of Metallurgy, Northeastern University, 3-11Wenhua Road, Heping District, Shenyang 110819, China; libo1310254@163.com (B.L.); gaobl@smm.neu.edu.cn (B.G.)

**Keywords:** coarse Al–Si alloy, selective liquation process, Zn–Al alloy, extraction process

## Abstract

A selective liquation process to extract Al from a coarse Al–Si alloy, produced by carbothermal reduction, was investigated on the laboratory scale. The products obtained by selective liquation–vacuum distillation were analyzed by X-ray diffraction, inductively coupled plasma optical emission spectrometry and scanning electron microscopy. During the selective liquation process with the use of zinc as the solvent, the pure aluminum in the coarse Al–Si alloy dissolved in the zinc melt to form an α-solid solution with zinc, and most of the silicon and iron-rich phases and Al–Si–Fe intermetallics precipitated and grew into massive grains that entered into the slag and separated with the Zn–Al alloy melt. However, some fine silicon particles remained in the Zn–Al alloy. Thus, Al–Si alloys conforming to industrial application standards were obtained when the Zn–Al alloys were separated by a distillation process.

## 1. Introduction

The production rate of global primary aluminum is second only to iron among metallic materials, and its output was more than sixty-five million tons in 2020. At present, aluminum is produced by the electrolysis of alumina, which is extracted from bauxite. As the only method of industrial production, electrolysis methods are high in cost, consume considerable amounts of energy, contribute to serious environmental pollution, and require high-grade bauxite [1,2]. More than four tons of high-grade bauxite releases more than four tons of red mud when one ton of aluminum is manufactured, and another 30 kg of spent potlining is released after electrolytic cell failure [3,4]. The red mud is a kind of highly alkaline slag [5,6], and spent potlining is a hazardous waste containing toxic and soluble cyanides and fluorides [7,8]. There are currently no effective methods for disposing of red mud and the spent potlining. At present, these waste products are stockpiled, which not only occupies land, but also raises a range of environmental issues. Furthermore, supplies of high-grade bauxite are insufficient and are being rapidly consumed. Environmental protection and effective use of limited resources are the main problem restricting the development of the aluminum industry. Many researchers are exploring new more efficient aluminum extraction methods [9,10,11,12].

There are a large number of low-grade alumina-containing ores and industrial aluminiferous waste slag. Most low-grade alumina-containing resources, such as low-grade bauxite, kaolinite, and feldspar, are mainly composed of Al_2_O_3_ and SiO_2_. Most industrial aluminiferous waste slag also mainly contains Al_2_O_3_ and SiO_2_, such as fly ash, aluminum ash, flotation tailings of bauxite, oil shale residue, and gangue. The waste slag causes serious pollution in the surrounding environment and requires effective processing

The production of a coarse Al–Si alloy by carbothermal reduction is considered to be one potential method for making use of industrial aluminiferous waste slag [13,14]. It is also considered to be an efficient method of extracting aluminum from low-grade aluminum ore. The underlying principle of the method is the carbothermal reduction of Al_2_O_3_ and SiO_2_ in an arc furnace. A coarse Al–Si alloy containing 35–60 wt% aluminum, 25–60 wt% silicon, and 2–15 wt% iron can be obtained after carbothermal reduction. When the content of iron is higher than 5 wt%, the material is also called a coarse Al–Si–Fe alloy. Coarse Al–Si alloys have several applications, such as deoxidizers for steelmaking and reductants for magnesium smelting, or can be used as materials for extracting aluminum and to produce casting Al–Si alloys [15]. Extracting aluminum from casting Al–Si alloys is considered to be a feasible process for aluminum production. The following methods are used to extract aluminum from coarse Al–Si alloys. The first involves disproportionation reactions of aluminum in the coarse Al–Si alloy or Al–Si–Fe alloy by reaction with AlCl_3_ or AlF_3_ to form AlCl or AlF gas at high temperature, which is separated from Si and Fe and reverted to aluminum, AlCl_3_ or AlF_3_ at low temperature [16,17]. The second method involves the separation of aluminum by molten salt electrolysis. The aluminum in the coarse Al–Si alloy as a soluble anode is dissolved into the electrolytes and reduced to aluminum at the cathode, whereas Si, Fe, and other impurities remain in the anode alloy in solid form. The third method is selective liquation, which takes advantage of the higher solubility of aluminum compared to iron and silicon in zinc, magnesium or mercury at a given temperature. Aluminum dissolved in zinc, magnesium, or mercury forms a liquid alloy, which separates from Si, Fe, and other impurities that form slag. Of these three methods for extracting aluminum from coarse Al–Si alloys, the third has the lowest energy consumption and highest efficiency.

In the present paper, the mechanism of the extraction process of aluminum and the distribution of Al, Si and Fe phases in alloys and Zn–Al–Si–Fe slags by the selective liquation of zinc were studied.

## 2. Materials and Methods

### 2.1. Materials

**Figure 1:**
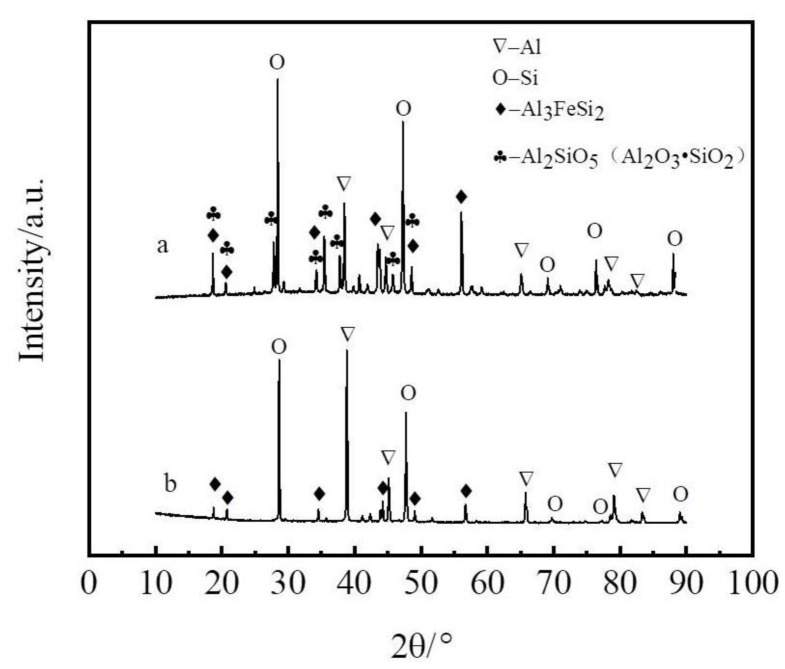
XRD of spectra of coarse Al–Si alloy. (**a**) before; (**b**) after the refining.

**Figure 2:**
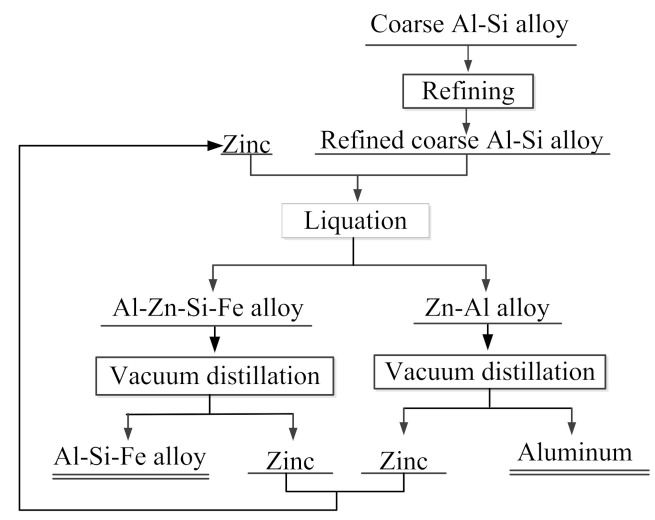
Extraction process flow diagram.

The coarse Al–Si alloys used in the experiments come from Dengfeng Electric Group Aluminum Alloy Corporation, Henan, China. The coarse Al–Si alloy used kaolinite as its raw material and anthracite as a reductant, and thus contained many metallic impurities, oxides and carbides. Iron, calcium, and titanium are the main metallic impurities. Other impurities include alumina, ferric oxide, and silicon oxide in the form of complex oxides such as porzite (3Al_2_O_3_∙2SiO_2_). The impurities in the form of oxides can be removed by refining, but the metallic impurities cannot. Figure 1 shows X-ray diffraction (XRD) of the coarse Al–Si alloy before and after refining. In the present experiments, a solvent refining method was used. A mixture of NaCl, KCl, and Na_3_AlF_6_ was used as the purificant. The NaCl and KCl used in the experiments were all analytically pure reagents, produced by Sinopharm Chemical Reagent Co., Ltd., (Beijing, China) where NaCl and KCl were dried at 300 °C for 5 h. Table 1 lists the main composition of the coarse Al–Si alloy and refining coarse Al–Si alloy. The Zn liquation agent had a purity of 99.9% and was acquired from Huludao Zinc Industry Co. Ltd., Liaoning, China. Figure 2 shows the extraction process flow diagram.

**Figure 3:**
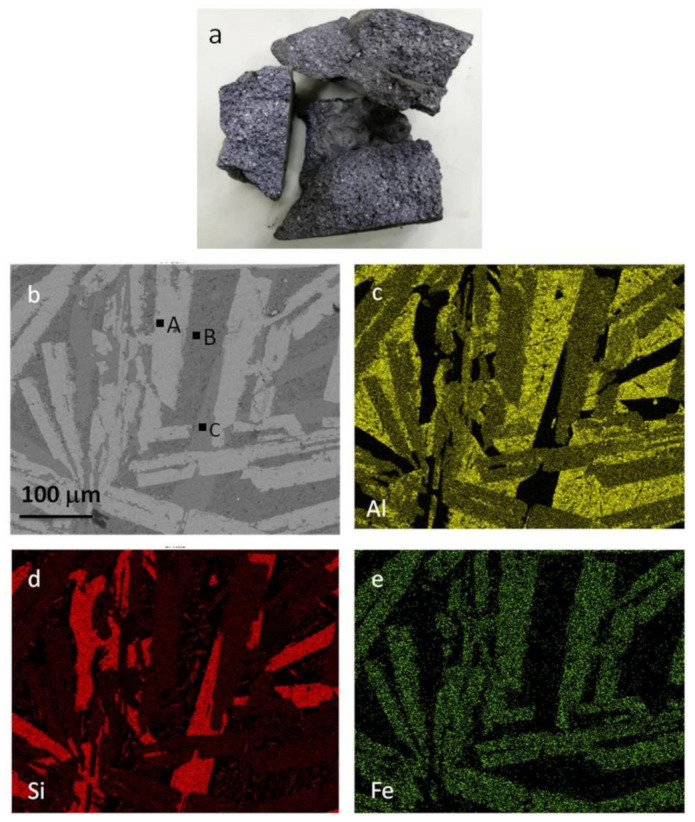
Photograph of coarse Al–Si alloy and elemental distributions of refined coarse Al–Si alloy. (**a**) coarse Al–Si alloy; (**b**) SEM of coarse Al–Si alloy; (**c**) Distribution of aluminum; (**d**) Distribution of silicon; (**e**) Distribution of iron.

As shown in Table 1, there is a high content of impurities in the coarse Al–Si alloy produced by the carbothermal reduction method. Figure 3b–e show the elemental distribution and scanning electron microscope images of the Al–Si alloy, respectively. The Fe-rich phases in the coarse Al–Si alloy are all in the form of Al–Si–Fe intermetallics (i.e., 39.43 wt% Al, 32.79 wt% Si, and 27.78 wt% Fe). As shown in Figure 3b, the aluminum is mainly in the form of pure aluminum (e.g., point B) and Al–Si–Fe intermetallics (e.g., point A), and the silicon is mainly in the form of pure silicon (e.g., point C) and Al–Si–Fe intermetallics (e.g., point A). In addition, a small amount of fine silicon particles is unevenly distributed in the aluminum with some dissolution into the aluminum. Refining experiments were carried out at 1187 K for 30 min using a composition of 47.0 wt%NaCl–9.0 wt%KCl–44.0 wt.% Na_3_AlF_6_ as the purificant. As shown in Figure 1, the diffraction peaks of many oxides are not obvious after the refining process.

### 2.2. Experimental Process

Experiments were performed in a high-purity alumina crucible with a capacity of 1000 g, an internal diameter of 100 mm, and a height of 150 mm. Before the liquation experiments, the zinc metal was placed into the alumina crucible and heated to 1223 K in a resistance furnace. The refined coarse Al–Si alloy was added to the zinc, and stirred slowly until all the coarse Al–Si alloy melted into the zinc to form a Zn–Al–Si–Fe alloy [18,19]. Afterwards, the alloy was slowly cooled to 823–1023 K and maintained at that temperature for 30 min. Some semi-solidified solid alloy, i.e., slag, separated out from the melt and collected under the melt. The semi-solidified solid slag was an Fe- and Si-rich Zn–Al–Si–Fe alloy, and the melt was a Zn–Al alloy. After the liquation process, vacuum distillation experiments were performed in a vacuum furnace. In the distillation process, the Zn was distilled from the Zn–Al alloy and the Zn–Al–Fe–Si alloy, aluminum, and an Al–Si–Fe alloy were obtained [20,21]. Most of the aluminum was extracted from the coarse Al–Si alloy by selective liquation and vacuum distillation.

The composition of alloys and slag was analyzed by inductively coupled plasma optical emission spectrometry (ICP-OES, iCAP 6500, Thermo Fisher Scientific, Waltham, MA, USA). The phases and microstructure of the products were identified by X-ray diffraction analysis (XRD, Bragg–Brentano mode, X Pert ProMPDDY2094 (PANalytical B.V., Almelo, The Netherlands), 2*θ*: 5–90°, step size: 0.015°/0.1 s, Working voltage: 40 kV, Working current: 40 mA) and scanning electron microscopy (SEM, Ultra Plus-43-13, Zeiss, Oberkochen, German). The yield of the Zn–Al alloy of the selective liquation process was calculated by Equation (1) and the Al extraction efficiency was calculated by Equation (2):

(1) $$\eta_{\mathrm{Zn}-\mathrm{Al}}=\frac{m_{\mathrm{Zn}-\mathrm{Al}}}{m_{\mathrm{Zn}-\mathrm{Al}}+m_{s}}\times 100\%$$

(2) $$\eta_{\mathrm{Al}}=\frac{m_{\mathrm{Zn}-\mathrm{Al}}\times \left(1-\omega_{\mathrm{Zn}}\right)}{M_{\mathrm{Al}-\mathrm{Si}}}\times 100\%$$

where $\eta_{\mathrm{Al}}$ is the Al extraction efficiency (%); $\eta_{\mathrm{Zn}-\mathrm{Al}}$ is the Zn–Al alloy extraction efficiency (%); $m_{\mathrm{Zn}-\mathrm{Al}}$ is the weight of the Zn–Al alloy (g); $m_{s}$ is the weight of slag (g); $\omega_{\mathrm{Zn}}$ is the content of Zn in the Zn–Al alloy (wt%); and $M_{\mathrm{Al}-\mathrm{Si}}$ is the weight of the coarse Al–Si alloy (g).

## 3. Results

### 3.1. Selective Liquation Process

#### 3.1.1. The Influence of Liquation Temperature

**Figure 4:**
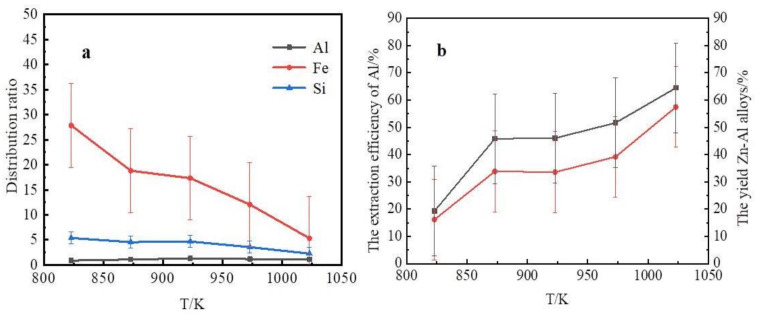
The influence of liquation temperature. (**a**) Curves of the extraction rate and the yield at various liquation temperatures; (**b**) The element distribution ratio of slag to alloy.

In the selective liquation process, the liquation temperature affects the quality of Zn–Al alloys and determines the content of silicon and iron in the Zn–Al alloy. The influence of the liquation temperature under conditions of a 2.5-mass ratio of zinc to coarse Al–Si alloy is shown in Figure 4.

**Figure 5:**
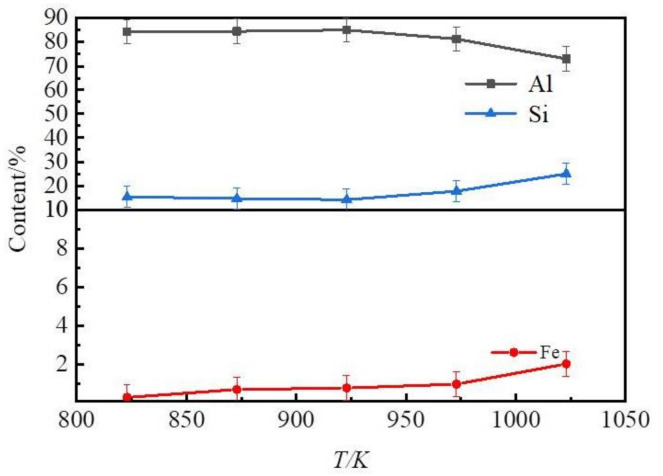
Effect of liquation temperature on composition of the Al–Si alloys.

Figure 4a shows the iron and silicon distribution ratio of slag to alloy obtained at different temperatures. This result shows that the contents of iron and silicon in slag were far higher than those in the Zn–Al alloys. After a selective liquation process, a Zn–Al alloy, which contained mainly Zn, Al, and Si, and a slag which contained mainly Zn, Al, Si, Fe, Ti, and Ca were obtained. Hence, most of the silicon and other impurities, such as iron, titanium, and calcium, in the coarse Al–Si alloy were separated from the aluminum by the selective liquation process. The iron and silicon distribution ratio of slag to alloy decreased as the liquation temperature was increased. Thus, an Al–Si alloy rather than pure aluminum was obtained after zinc was removed by vacuum distillation. According to Equation (1), the extraction efficiencies of aluminum and the yield of Zn–Al alloys at various liquation temperatures are shown in Figure 4b. This result shows that the extraction efficiency of aluminum and the yield of Zn–Al alloys increased as the liquation temperature was increased. When the zinc of the Zn–Al alloy and slag was distilled out, Al–Si alloys and Al–Si–Fe alloys were obtained. The contents of Si, Fe, and Al in the Al–Si alloys are plotted in Figure 5.

As shown in Figure 5, the content of silicon and iron in Al–Si alloys increased. The contents of Al, Si, and Fe in the Al–Si alloys were 73–85 wt%, 15–25 wt% and 0.30–2.00 wt%, respectively. When the temperature was below 873 K, the content of Fe in the products obtained by the distillation of Zn–Al alloys was lower than 0.70 wt%. The contents of other impurities conformed with the quality standards of the casting Al–Si alloys. When the liquation temperature was greater than 873 K, the content of Fe surpassed the ratified content (0.7 wt%).

#### 3.1.2. The Influence of the Mass Ratio of Zinc to Coarse Al–Si Alloy 

**Figure 6:**
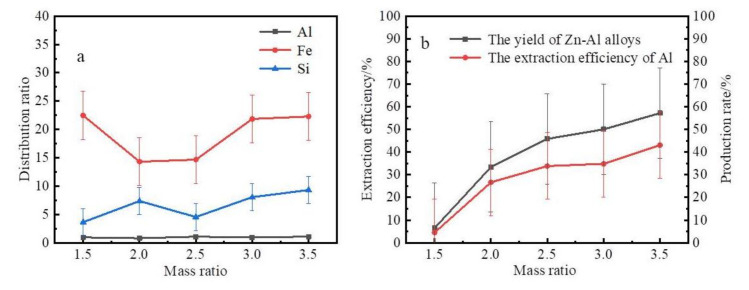
The influence of the mass ratio of zinc to coarse Al–Si alloy. (**a**) Effect of mass ratio on the extraction rate and yield. (**b**) The element distribution ratio of slag to alloy.

The mass ratio of zinc to coarse Al–Si alloy is another factor that affects the extraction of Al from coarse Al–Si alloys. The effects of the mass ratio of zinc to coarse Al–Si alloy on the slag and product contents when the liquation temperature was 873 K are shown in Figure 6.

**Figure 7:**
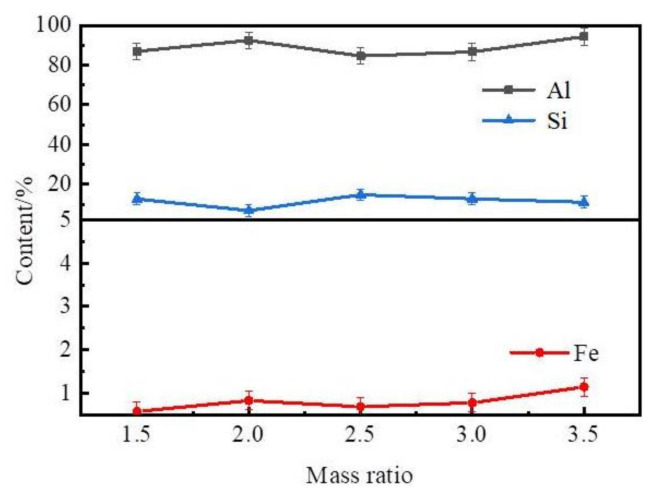
Effect mass ratio on composition of the Al–Si alloys.

Figure 6a shows that the iron and silicon distribution ratio of slag to alloy was high at different mass ratios of zinc to coarse Al–Si alloy. As shown in Figure 6b, the extraction efficiency of aluminum and the yield of the Zn–Al alloys increased as the mass ratio of zinc to coarse Al–Si alloy was increased. The content of Al and Si in the Zn–Al alloys decreased as the mass ratio of zinc to coarse Al–Si alloy was increased. The contents of Si, Fe, and Al in the Al–Si alloys were distilled out and are plotted in Figure 7.

These results show that the content of Al and Si in the Al–Si alloys was in the range of 84–94 wt% and 6–15 wt%. The content of Fe in the Al–Si alloys was in the range of 0.50–1.20 wt% and increased as the mass ratio of zinc to coarse Al–Si alloy was increased. 

**Figure 8:**
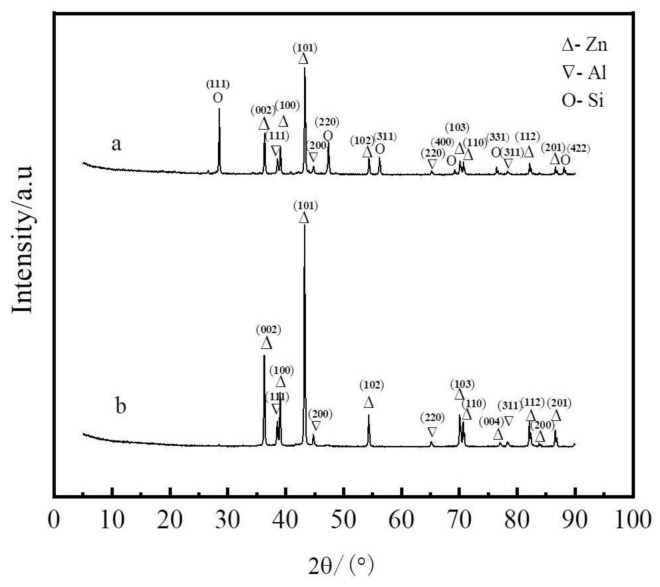
XRD patterns of the Zn–Al alloy and slag. (**a**) Slag; (**b**) Zn–Al alloy.

Figure 8 shows XRD patterns of the Zn–Al alloy and slag at a liquation temperature of 873 K and 2.5 mass ratio of zinc to coarse Al–Si alloy.

Therefore, Al–Si alloys rather than pure aluminum are formed by a selective liquation process involving coarse Al–Si alloys as a feedstock and using zinc as a solvent. The Al–Si alloys can be used as master alloys for casting Al–Si alloys. Among the requirements for casting Al–Si alloys, Fe is the primary impurity and its content should be below 0.7 wt%. To meet these iron requirements, the liquation temperature should be maintained below 923 K and the mass ratio of zinc to coarse Al–Si alloy should be below 3.0. The optimum technological conditions are a liquation temperature of 873–925 K and a mass ratio of zinc to Al–Si alloy in the range of 2.5–3.0. Under these conditions, the Al–Zn alloy contains approximately 80 wt% zinc, 16–17 wt% aluminum, 2–3 wt% silicon, and less than 0.17 wt% iron. When the zinc is distilled out, the Si content in Al–Si alloys was 10–15 wt%, the Fe content was below 0.7 wt%, and contents of other impurities were very low.

### 3.2. Principle of Selective Liquation Process with Zinc

**Figure 9:**
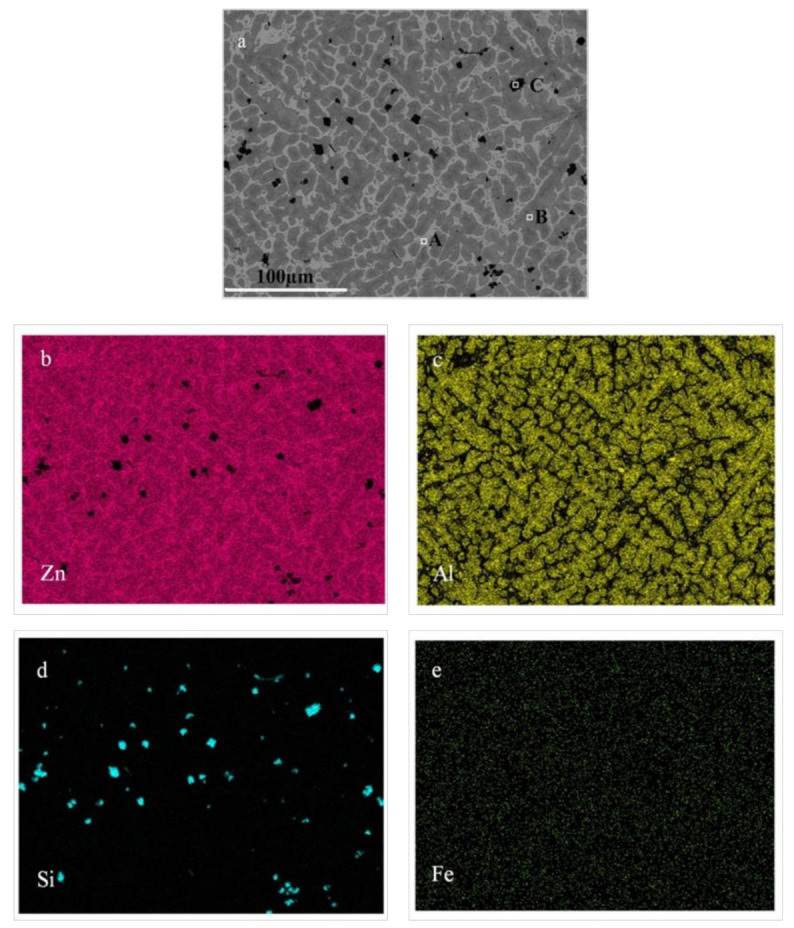
Elemental distributions of Zn–Al alloy. (**a**) SEM of Zn–Al alloy; (**b**) Distribution of zinc; (**c**) Distribution of aluminum ; (**d**) Distribution of silicon ; (**e**) Distribution of iron.

**Figure 10:**
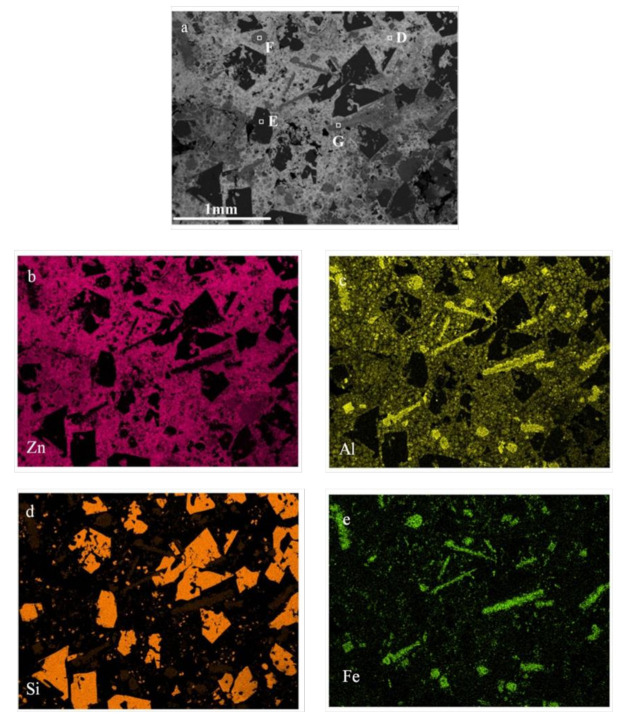
Elemental distributions of Zn–Al–Si–Fe alloy slag. (**a**) SEM of Zn–Al–Si–Fe alloy; (**b**) Distribution of zinc; (**c**) Distribution of aluminum; (**d**) Distribution of silicon; (**e**) Distribution of iron.

The SEM and elemental distribution of the Zn–Al alloy and slag formed by selective liquation at 873 K with a 2.5 mass ratio of zinc to coarse Al–Si alloy are shown in Figure 9 and Figure 10, respectively.

The SEM, elemental distribution and EDS analyses indicate that the Zn–Al alloy mainly consisted of a η-solid solution (e.g., point A, consisting of 100% Zn) and an α-solid solution [22] (e.g., point B, approximately 75% Zn and 25% Al). Except for a very small amount in the solid solution, most of the silicon was in the form of pure silicon grains (e.g., point C, which is 100% Si). The iron content was very low and evenly distributed throughout the alloy.

The SEM, elemental distribution and EDS analyses indicate that the slag is a kind of Zn–Al–Si–Fe alloy and mainly consists of a η-solid solution (e.g., point D consists of 98.74% Zn and 1.26% Al), pure silicon grains (e.g., point E consists of 100% Si), and Al–Si–Fe intermetallics. Most of the silicon is in the form of large pure silicon grains and some is in the form of Al–Si–Fe intermetallics. The iron-rich phase is an all Al–Si–Fe intermetallic, which is granular (e.g., point F and consists of 39.91% Al, 33.03% Si, 25.16% Fe, and 1.9% Zn) and a long strip (e.g., point G consists of 23.31% Al, 39.87% Si, 32.46% Fe, and 4.19% Zn).

On the basis of the results presented in Figure 3, Figure 9 and Figure 10, it is concluded that during the selective liquation process, the pure aluminum in the coarse Al–Si alloy enters into the zinc melt to form an α solid solution. The silicon existing in the form of pure silicon grows into massive silicon grains, most of which deposit into the slag at the bottom of melt. However, some fine silicon particles remain in the Zn–Al alloy. The iron-rich phases, i.e., the Al–Si–Fe intermetallics, all deposit into the slag and some become needle-like phases with high iron contents during the selective liquation process. The iron content of the obtained Zn–Al alloy is very low (below 0.20 wt%).

### 3.3. Zinc Separated by a Distillation Process

The zinc in the Zn–Al alloy and slag can be separated by a distillation process when the distillation temperature is greater than 1173 K. The zinc can be recovered and an Al–Si alloy is obtained. The ICP results of the Al–Si alloy indicate that the content of zinc is less than 0.1%. Hence, zinc is effectively completely recovered and might be reused. Table 2 lists the main composition of the Al–Si alloy after zinc is separated by a distillation process. 

## 4. Conclusions

In the coarse Al–Si alloy produced by a carbothermal reduction method, Fe-rich phases are present in the form of Al–Si–Fe intermetallics, and silicon is present mainly in the form of pure silicon and Al–Si–Fe intermetallics. During a selective liquation process, pure aluminum in the coarse Al–Si alloy dissolves in the zinc melt to form an α-solid solution with zinc, and most of the silicon and the iron-rich phases, Al–Si–Fe intermetallics, precipitate and grow into massive grains, which deposit into the slag and separate with the Zn–Al alloy melt; however, some fine silicon particles remain in the Zn–Al alloy. The product of the aluminum extraction from a coarse Al–Si alloy by selective liquation with zinc as the solvent is an Al–Si alloy with a low content of Fe rather than pure aluminum. A kind of Al–Si alloy conforming to industrial application standards, containing 85–90 wt% Al, 10–15 wt% Si, and less than 0.6 wt% Fe, can be obtained at a liquation temperature of 873 K and a 2.5 mass ratio of zinc to the coarse Al–Si alloy. The aluminum extraction rate is in the range of 35–40%.

## Figures and Tables

**Table 1:** Composition of the coarse Al–Si alloy (wt%).

Element	Al	Si	Fe	Ca	Ti	Mg	Zn	Cu	Other Impurities
Coarse alloy	58.50	25.48	4.36	2.06	0.72	0.01	0.01	0.01	<12.35
Refining coarse alloy	60.50	31.35	5.31	1.87	0.94	0.01	0.01	0.01	-

**Table 2:** Composition of Al–Si alloy (wt%).

Element	Al	Si	Fe	Zn	Ti	Other Impurities
Content	84.13	14.59	0.58	0.10	<0.1	<0.5

## Data Availability

Data are contained within the article.
